# Supplementary material for: Automation assisted anaerobic phenotyping for metabolic engineering
Source: Microb Cell Fact. 2021 Sep 23;20:184. doi: 10.1186/s12934-021-01675-3 (PMC8461876; doi:10.1186/s12934-021-01675-3)
Supplement: Supplementary file 1 — Additional file 1: Table S1. Names and formulae of reaction abbreviations mentioned in Fig. 7 (main text). Metabolites in the reaction formulae are represented by their BiGG ID. Figure S1. Change in sterility with air-gap (data consolidated from main text Fig. 2d). Figure S2. Increase in pipetting error upon increasing pipetting speed by 300% for different volume ranges. Figure S3. Distribution of biomass yields (ratio of final to initial biomass) of wild type E. coli MG1655 grown in Rich Defined Media with different seals. Figure S4. Time-course showing cell density and instantaneous growth rate of different E. coli strains (described in Materials & methods) in RDM with and without a layer of oil in the presence of oxygen and with a layer of mineral oil inside an anaerobic chamber. Figure S5. Time-course showing cell density and instantaneous growth rate of E. coli strains (described in Materials & methods) with different pre-culturing strategies. Figure S6. Variance explained by each principal component for principal component analysis performed on metabolite yields and growth rates of E. coli strains (described in Materials & methods) grown in rich defined media in a bioreactor and microplates supplemented with reducing agents. Figure S7. Principal component analysis performed on the metabolite yields and growth rates of E. coli strains (described in Materials & methods) grown in rich defined media in a bioreactor and microplates supplemented with reducing agents. Figure S8. A comparison E. coli's metabolite yields and growth rates obtained from a bench-top 0.5 L bioreactor and 96-well microplates with different reducing agents for the strains: a. Wild Type MG1655, b. MG1655 Δ(adhE; pta)-D1, c. MG1655 Δ(adhE; pta)-D28, and d. MG1655 Δ(adhE; pta)-D59. Figure S9. Principal component analysis performed on the metabolite yields and growth rates of E. coli strains (described in Materials & methods) grown in rich defined media in a bioreactor and microplates supplement [file 12934_2021_1675_MOESM1_ESM.pdf]

# Additional File 1

**Table S1:** Names and formulae of reaction abbreviations mentioned in Figure 7 (main text). Metabolites in the reaction formulae are represented by their BiGG ID

| Abbreviation | Reaction Name | Reaction Formula |
|---|---|---|
| ack | Acetate kinase | ac_c + atp_c <=>actp_c + adp_c |
| adh | Alcohol/aldehyde dehydrogenase | acald_c + coa_c + nad_c <=>accoa_c + h_c + nadh_c<br>etoh_c + nad_c <=>acald_c + h_c + nadh_c |
| frd | Fumarate reductase | fum_c + mql8_c ->mqn8_c + succ_c |
| fum | Fumarase | fum_c + h2o_c <=>mal__L_c |
| ldh | Lactate dehydrogenase | lac__D_c + nad_c <=>h_c + nadh_c + pyr_c |
| mdh | Malate dehydrogenase | mal__L_c + nad_c <=>h_c + nadh_c + oaa_c |
| pfl | Pyruvate formate lyase | coa_c + pyr_c ->accoa_c + for_c |
| pox | Pyruvate oxidase | h2o_c + pyr_c + q8_c ->ac_c + co2_c + q8h2_c |
| ppc | Phosphoenolpyruvate carboxylase | co2_c + h2o_c + pep_c <=>h_c + oaa_c + pi_c |
| pta | Phosphotransacetylase | accoa_c + pi_c <=>actp_c + coa_c |

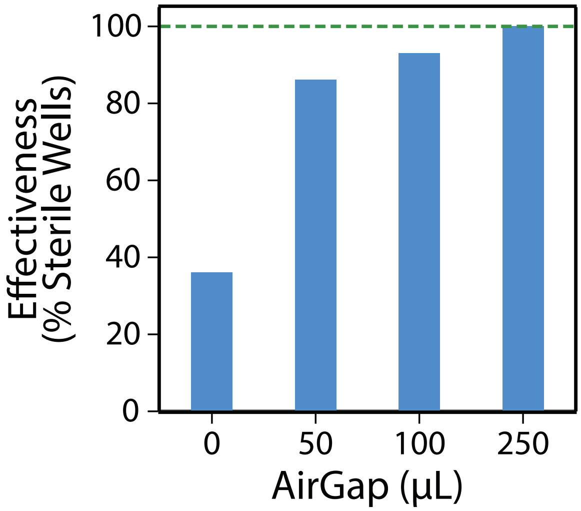


**Figure S1:** Change in sterility with air-gap (data consolidated from main text Figure 2d).

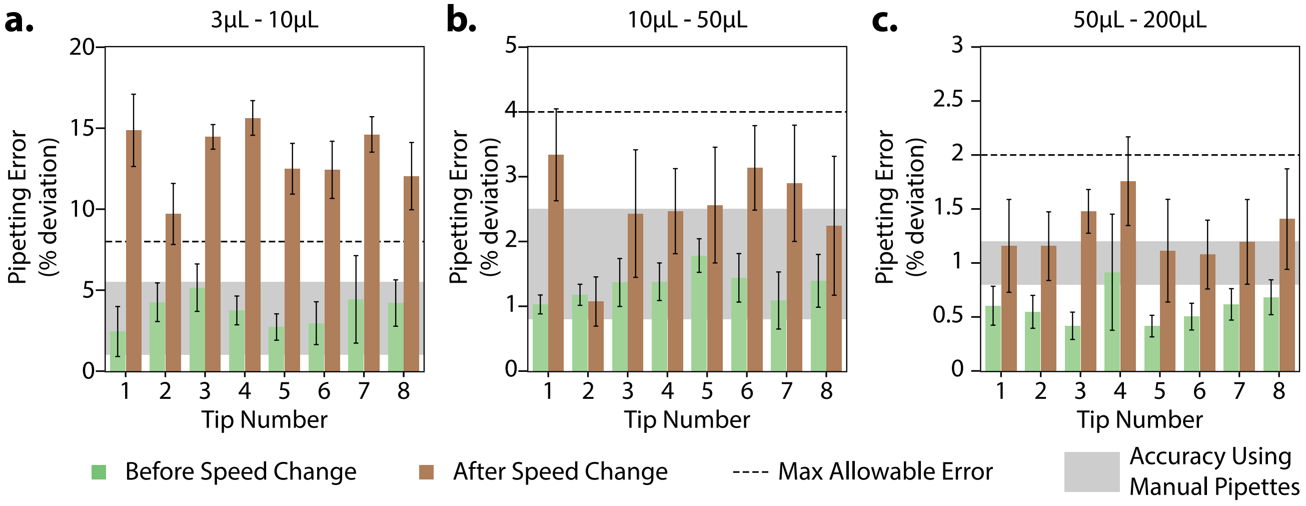


**Figure S2:** Increase in pipetting error upon increasing pipetting speed by 300% for different volume ranges.

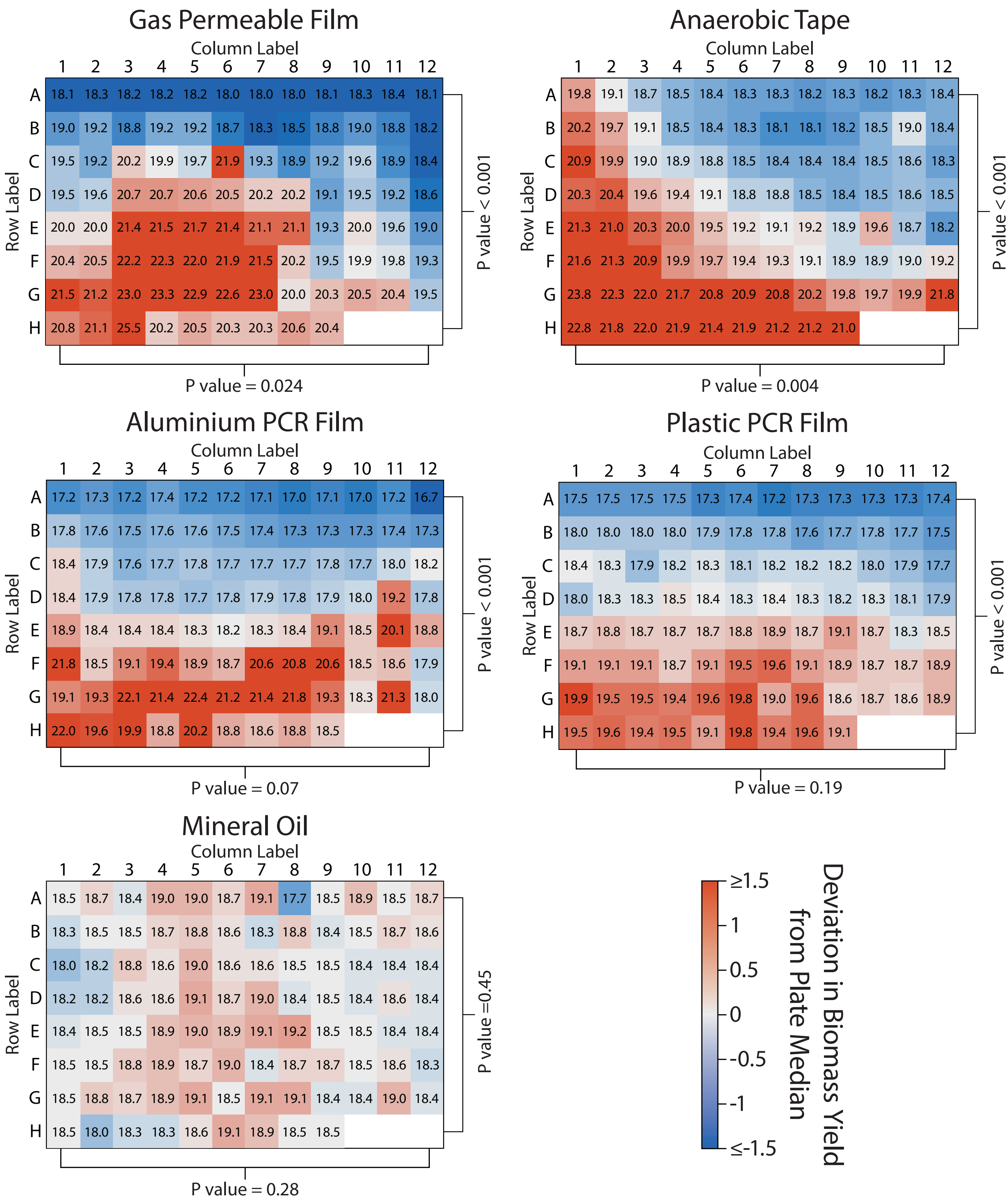


**Figure S3:** Distribution of biomass yields (ratio of final to initial biomass)of wild type *E. coli* MG1655 grown in Rich Defined Media with different seals. Yield values recorded in each well of the plates are shown, with a heatmap illustrating the deviation of the yield from the median value of the plate. P values for a two-tailed t-test with the null hypothesis that the first and last column/row have no significant differences in average biomass yields are shown.

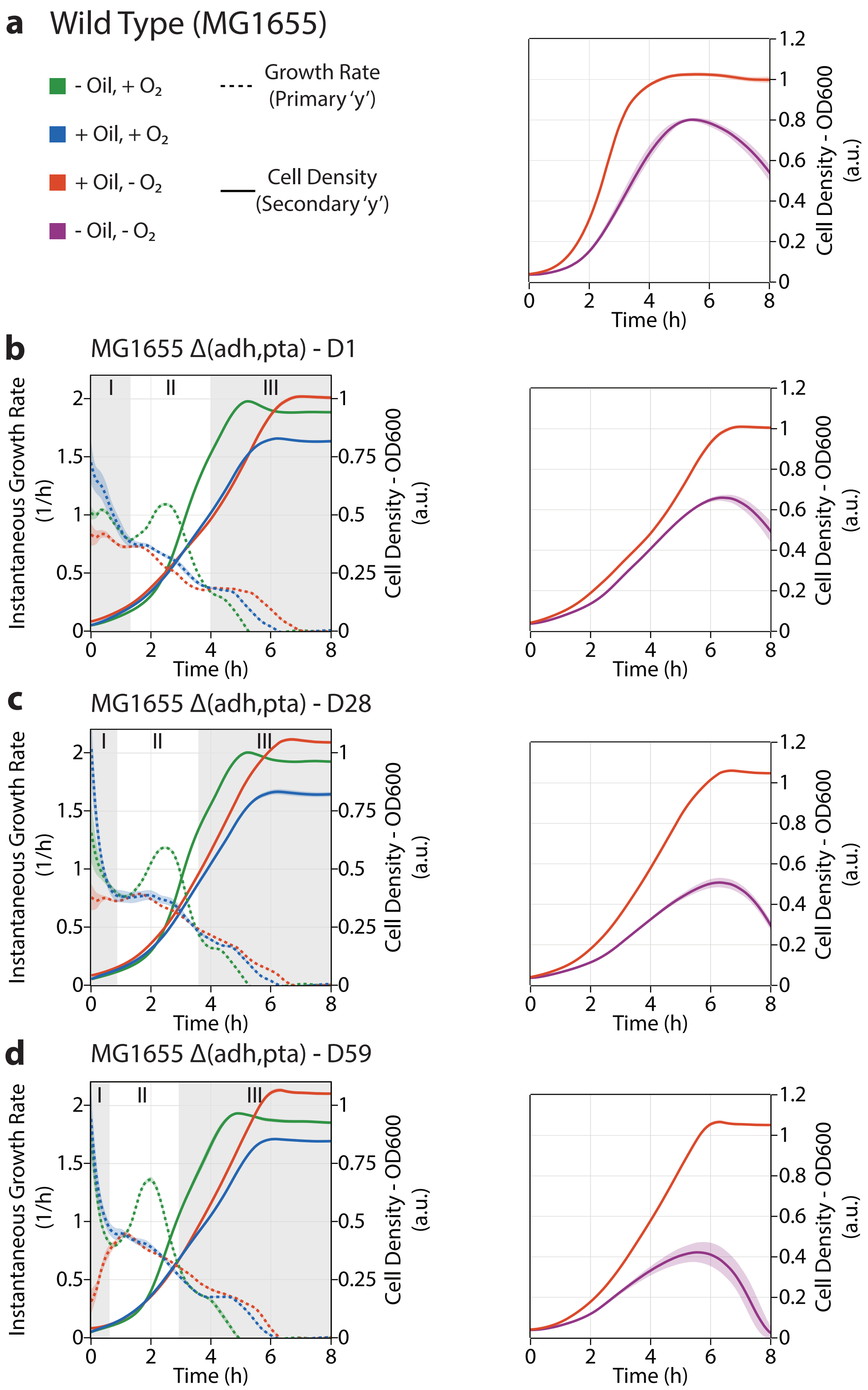


**Figure S4:** Time-course showing cell density and instantaneous growth rate of different *E. coli* strains (described in Materials & Methods) in RDM with and without a layer of oil in the presence of oxygen and with a layer of mineral oil inside an anaerobic chamber. Decreaase in absorbance of strains grown anaerobically without the oil is due to evaporation of culture media.

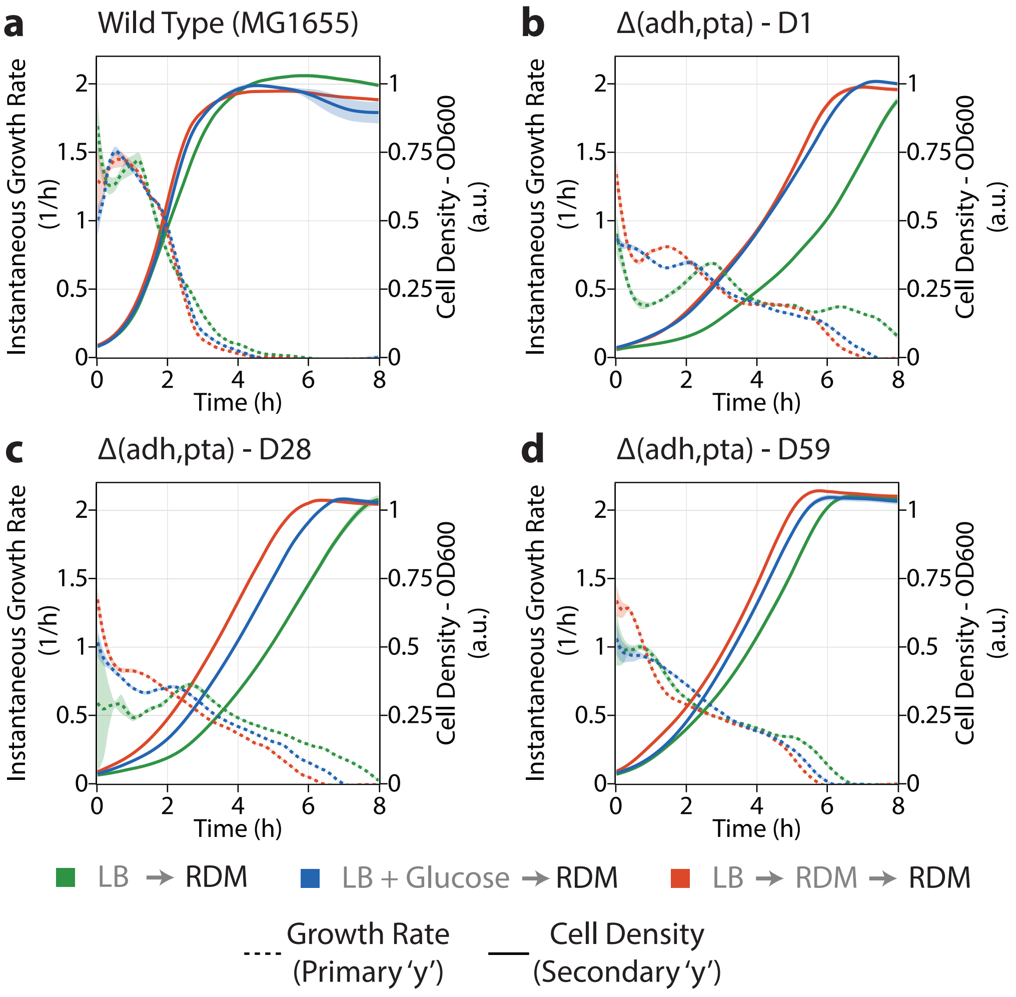


**Figure S5:** Time-course showing cell density and instantaneous growth rate of *E. coli* strains (described in Materials & Methods) with different pre-culturing strategies. For all strains, cells transferred from LB+glucose to RDM showed similar growth profiles to those with an intermediate adaptation transfer to RDM. In contrast, cells transferred from LB to RDM directly showed a longer lag phase and slower growth in all cases.

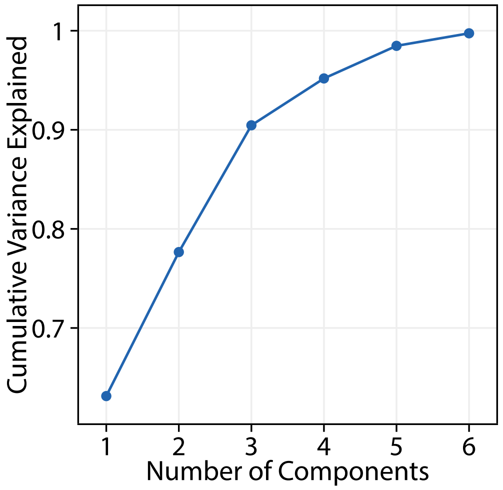


**Figure S6:** Variance explained by each principal component for principal component analysis performed on metabolite yields and growth rates of *E. coli* strains (described in Materials & Methods) grown in rich defined media in a bioreactor and microplates supplemented with reducing agents.

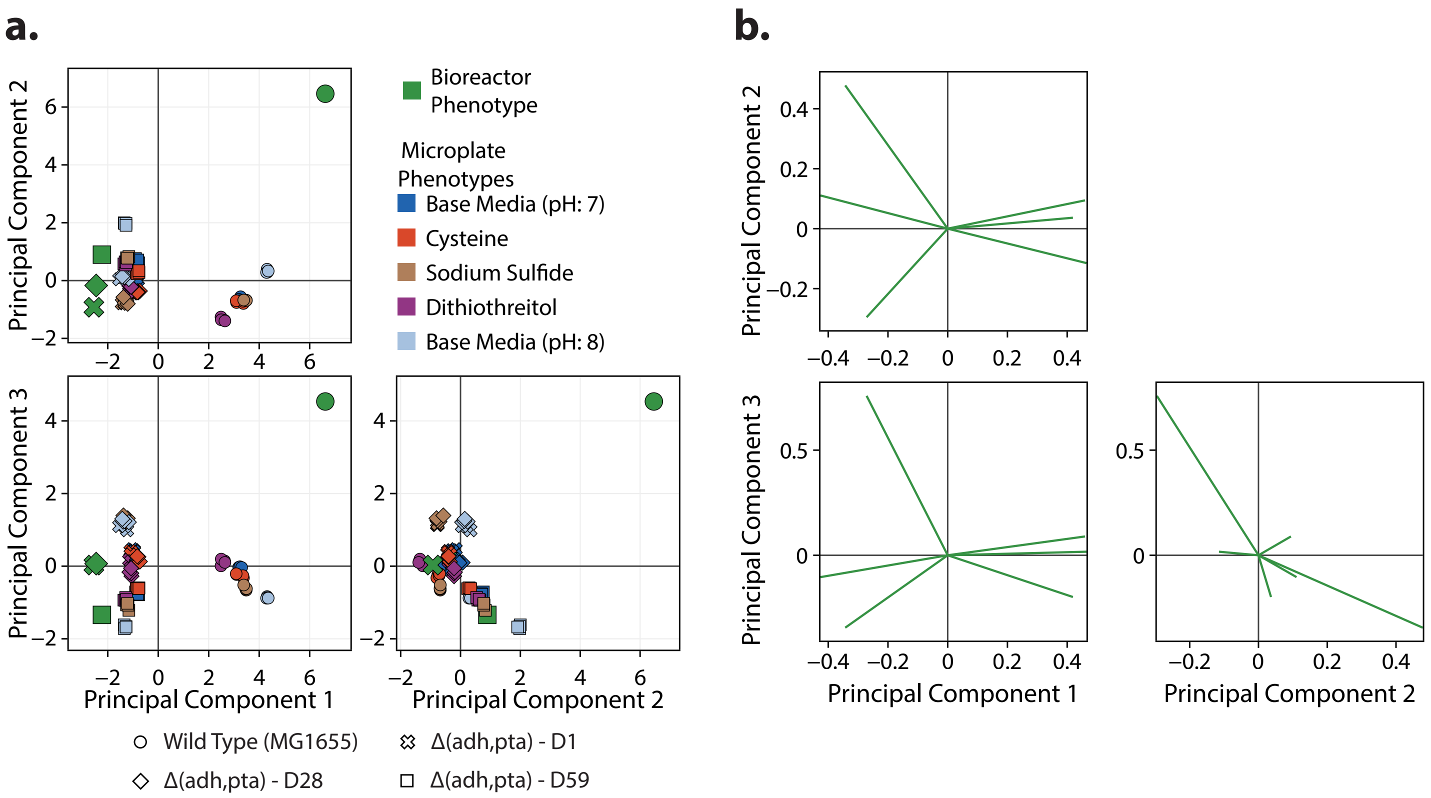


**Figure S7:** Principal component analysis performed on the metabolite yields and growth rates of *E. coli* strains (described in Materials & Methods) grown in rich defined media in a bioreactor and microplates supplemented with reducing agents. **a.** Scores and **b.** Loadings of each feature from PCA analysis.

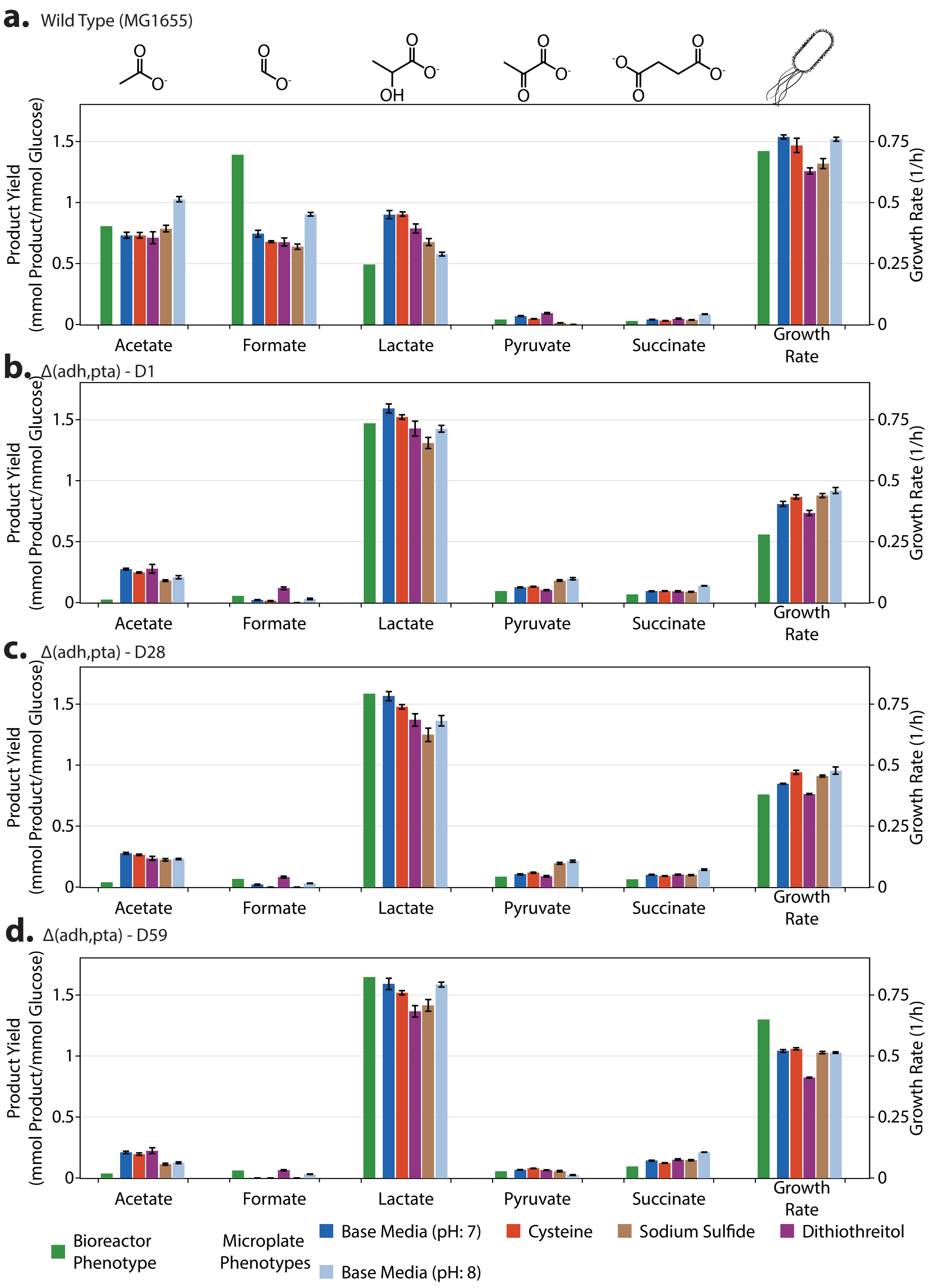


**Figure S8:** A comparison *E. coli*'s metabolite yields and growth rates obtained from a bench-top 0.5 L bioreactor and 96-well microplates with different reducing agents for the strains: **a.** Wild Type MG1655, **b.** MG1655 $\Delta(adhE, pta)$-D1, **c.** MG1655 $\Delta(adhE, pta)$-D28, and **d.** MG1655 $\Delta(adhE, pta)$-D59

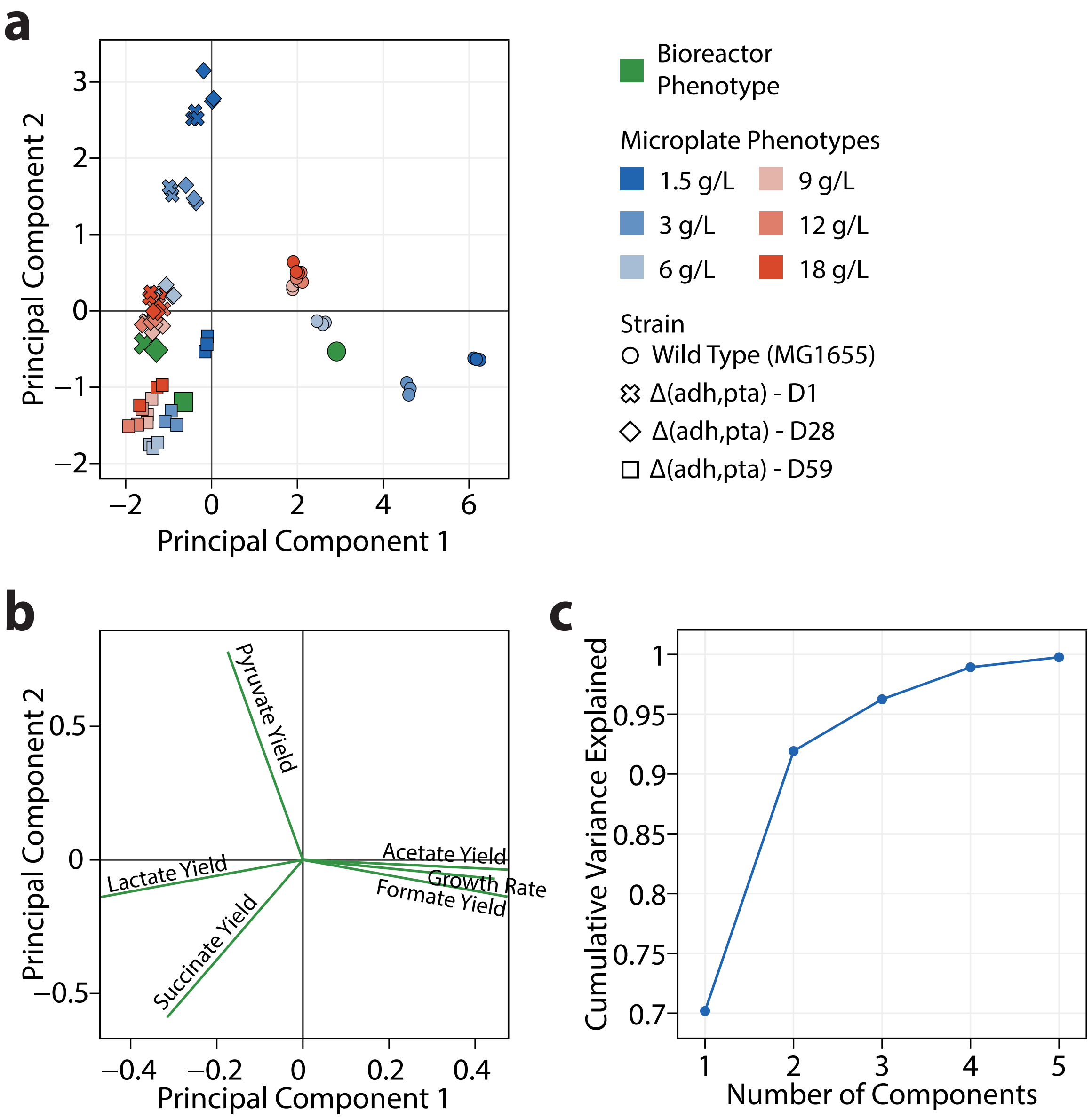


**Figure S9:** Principal component analysis performed on the metabolite yields and growth rates of *E. coli* strains (described in Materials & Methods) grown in rich defined media in a bioreactor and microplates supplemented with different substrate concentrations. **a.** Scores and **b.** Loadings of each feature from PCA analysis. **c.** Ratio of variance explained by each principal component.

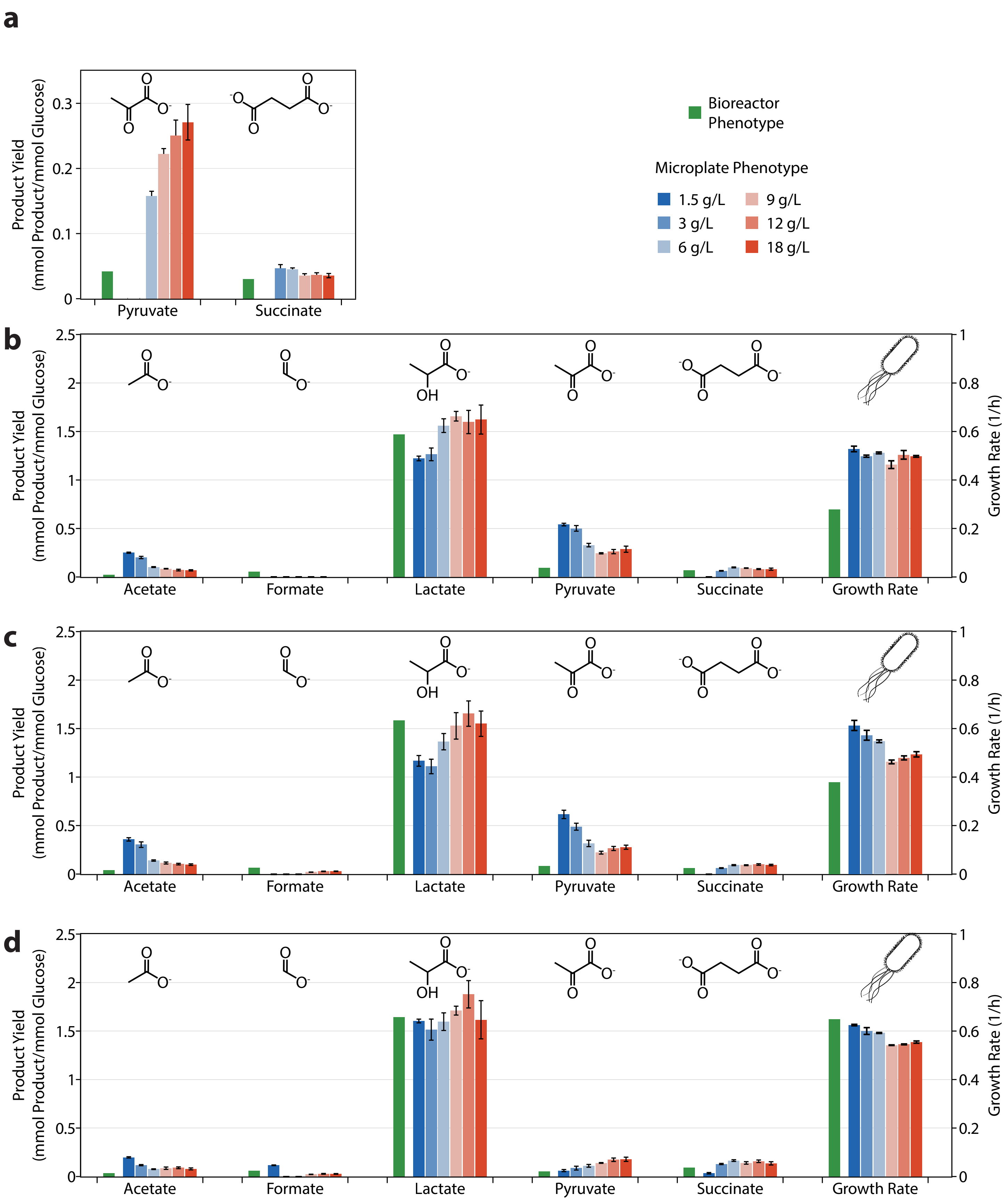


**Figure S10:** A comparison *E. coli*'s metabolite yields and growth rates obtained from a bench-top 0.5 L bioreactor and 96-well microplates with different initial glucose concentrations for the strains: **a.** Wild Type MG1655, **b.** MG1655 $\Delta(adhE, pta)$-D1, **c.** MG1655 $\Delta(adhE, pta)$-D28, and **d.** MG1655 $\Delta(adhE, pta)$-D59
